# EEF1D Promotes Glioma Proliferation, Migration, and Invasion through EMT and PI3K/Akt Pathway

**DOI:** 10.1155/2020/7804706

**Published:** 2020-09-24

**Authors:** Cheng Xie, Mingfeng Zhou, Jie Lin, Zhiyong Wu, Shengfeng Ding, Jie Luo, Zhengming Zhan, Yonghua Cai, Shuaishuai Xue, Ye Song

**Affiliations:** ^1^Department of Neurosurgery, Nanfang Hospital, Southern Medical University, Guangzhou, 510515 Guangdong, China; ^2^Department of Neurosurgery, The Second Affiliated Hospital of the Chinese University of Hong Kong (Shenzhen), Shenzhen, 518116 Guangdong, China

## Abstract

Eukaryotic translation elongation factor 1*δ* (EEF1D), a subunit of the elongation factor 1 complex of proteins, mediates the elongation process of protein synthesis. Besides this canonical role, EEF1D was found overexpressed in many tumors, like hepatocarcinomas and medulloblastomas. In the present study, we demonstrated for the first time that EEF1D may interact with other putative proteins to regulate cell proliferation, migration, and invasion through PI3K/Akt and EMT pathways in glioma. Furthermore, knockdown of EEF1D could reduce cell proliferation and impaired epithelial-mesenchymal transition (EMT) phenotypes, including cell invasion. Taken together, these results indicate that EEF1D and its partner proteins might play a critical role in glioma and serve as a potential therapeutic target of glioma.

## 1. Introduction

Glioma, the most common and malignant primary brain tumor, is characterized by high-grade proliferation, invasion, and poor prognosis. Being an aggressive tumor, glioma also shows poor response to common therapeutic treatments including surgery, radiation, and conventional concomitant and adjuvant chemotherapy with temozolomide (TMZ). Although treatment methods advanced during the past decades, the median survival of glioma is still about 14.6 months and five-year survival is less than 10% [1–4]. Considering that the molecular mechanism underlying glioma is still ambiguous, it is an urgent need to unravel mechanisms of proliferation, invasion, and resistance to identify weaknesses that may be exploited using new and existing agents to increase survival from glioma.

EEF1D, as a part of the eukaryotic translation elongation factor 1 (EEF1) complex, serves as the enzymatic delivery of aminoacyl tRNAs to the ribosome and functions as a guanine nucleotide exchange factor. Based on the canonical and noncanonical functions [5, 6], emerging amounts of evidence indicate that EEF1 proteins, particularly the prototypical member EEF1D, may play a role in the control of cellular processes during tumorigenesis [6]. In 2000, Shuda et al. reported a higher level of EEF1D mRNA in hepatocarcinomas [7], and in 2008, Piltti et al. reported the correlation of EEF1D with phosphorylation of ERK of the MAP kinase pathway in chondrosarcoma [8]. Later, with advances in detection approaches, such as chromosomal comparative genomic hybridization, two-dimensional electrophoresis coupled with MALDI–TOF-MS and quantitative PCR, the upregulation of EEF1D was described by several researchers in other cancers like esophageal carcinomas [9], non-small-cell lung cancers [10], and medulloblastomas [11]. However, little has been uncovered regarding the underlying biological mechanisms correlated with EEF1D overexpression in glioma, and the involvement of EEF1D in glioma tumorigenesis has not yet been investigated.

In the present study, we found that EEF1D may play a role in the development of glioma through multiple pathways and provided new insights into the glioma initiation. We also speculated that EEF1D may be involved in multiple major cell signaling pathways simultaneously like EMT and PI3K/Akt pathways to regulate cell survival, migration, and invasion in glioma. Furthermore, EEF1D may constitute a new prospect for a therapeutic target against human glioma.

## 2. Materials and Methods

### 2.1. Bioinformatics Analysis

The genomic expression data and clinicopathological data of 163 GBM tissues, 518 LGG tissues, and 207 normal brain tissues were downloaded from The Cancer Genome Atlas (TCGA, https://tcga-data.nci.nih.gov/TCGA). The putative EEF1D-interacting proteins were subjected to functional annotation analysis using FunRich analysis tool [12, 13].

### 2.2. Cell Culture

The U87 and A172 glioblastoma cell lines were purchased from the American Type Culture Collection (ATCC, USA). All cell lines were cultured in Dulbecco's modified Eagle's medium (DMEM) (Hyclone, Logan, USA) supplemented with 10% fetal bovine serum (FBS, Hyclone, USA) and incubated in a humidified atmosphere under 5% CO_2_ at 37°C.

### 2.3. Collection of Clinical Samples

Clinical sample tissues were collected from patients afflicted with glioma. All samples were then confirmed pathological diagnosis and classified according to the World Health Organization (WHO) criteria. Every human tissue was obtained with advanced written informed consent from patients or their guardians before participation in the study, and approval from the Ethics Committees of Nanfang Hospital of Guangdong Province was obtained.

### 2.4. Western Blot

Western blot was performed as previously described. The cells or clinical tissues were washed thoroughly with PBS three times and lysed with RIPA Buffer (50 mM Tris-HCl pH 8.0, 1 mM EDTA pH 8.0, 5 mM DTT, 2% SDS) with a protease inhibitor and phosphoric-acid protease inhibitor under 4°C for 30 minutes. The protein concentration was then determined using BCA assay (Beyotime Inc., China). The proteins were separated subsequently using SDS-PAGE gel and electro-transferred to polyvinylidene difluoride membranes (Invitrogen, Carlsbad, CA). The membranes were blocked with 5% BSA and then incubated with primary antibodies, including EEF1D (PTG, USA); E-cadherin, N-cadherin, *β*-catenin, slug, snail, and vimentin (Cell Signaling Technology, USA); PI3K and PI3K (phosopho-Tyr467/199); Akt and p-Akt (phosphor-Ser473) (SAB, USA); and GAPDH (CWbio, China), overnight at 4°C and then incubated with horseradish peroxidase-conjugated secondary antibody for 1 h under room temperature. Finally, signals were detected using enhanced chemiluminescence reagents (Pierce, Rockford, IL, USA). All experiments were independently performed in triplicate.

### 2.5. IHC

For IHC, tissue sections underwent gradient ethanol dehydration and infiltration of histological samples in tissue processor, were embedded in paraffin and 4 *μ*m thin sectioned, and were then deparaffinized in xylene. After washing thoroughly with PBS, the slides were treated with 10 mM citrate buffer for 2 minutes at 100°C to achieve antigen retrieval and treated with 3% hydrogen peroxide to block endogenous peroxidase activity. After washing thoroughly with PBS, slides were blocked with 5% BSA for 1 hour at room temperature and incubated with EEF1D primary antibody (PTG, USA) at 4°C overnight. After washing thoroughly with PBS, the slides were incubated with biotin-labeled goat anti-mouse or anti-rabbit antibodies (ZSGBbio, China) for 1 hour at room temperature. The sections were finally visualized with DAB, counterstained with hematoxylin, mounted in neutral gum, and imaged using a bright field microscope equipped with a digital camera (Leica, Germany).

### 2.6. Small Interfering RNA (siRNA) Transfection

Transfection of siRNA was performed according as previously described [14]. For EEF1D knockdown, siRNA targeting EEF1D along with the negative control was designed and synthesized by RiboBio (Guangzhou, China). The sequences to EEF1D were GCCGCAACAUCUUAGGGAA (siEEF1D-1), GCAACAUCUUAGGGAACAA (siEEF1D-2), and CCUUGCCCUACUGUUACUU (siEEF1D-3). Among three siRNAs targeting on EEF1D gene, the most effective one (siEEF1D) was identified by Western blot and applied for the further experiments. Glioma cells U87 and A172 were plated onto a 6-well plate at 30-50% confluence. After incubation for 6 hours at 37°C in a 5% CO_2_ atmosphere, siEEF1D mixed with Lipo2000 (Thermo Fisher, USA) was then transfected into cells following the manufacturer's protocol. Cells were then collected after 24-48 hours for further experiments.

### 2.7. Transwell and Boyden Assays

In vitro cell migration Transwell assay and invasion Boyden assay were performed according as previously described [14]. For the Transwell assay, 1 × 10^4^ cells in 100 *μ*l DMEM medium without FBS were seeded onto the upper chamber of the Transwell apparatus (Costar, MA), and 500 *μ*l DMEM with 10% FBS was added into the lower chamber as a chemoattractant. After incubation for 6 hours at 37°C in a 5% CO_2_ atmosphere, the fibronectin-coated polycarbonate membrane insert was washed with PBS, and cells adhering to the top surface of the insert were removed with a cotton swab. Cells on the lower surface were then fixed with methanol, stained with crystal violet solution, and counted under a microscope in five predetermined fields (200x). All assays were independently repeated at least thrice. For the Boyden assay, the procedure was similar to the above one, except for the fact that the Transwell membranes were first precoated with 24 *μ*g/*μ*l Matrigel (R&D Systems, USA), and the cells were incubated in the Transwell apparatus for 8 hours at 37°C in a 5% CO_2_ atmosphere. Cells on the lower surface were counted in the same way as the cell migration assay.

### 2.8. Immunoprecipitation

For immunoprecipitation, glioma U87 cells were rinsed once with cold PBS and lysed with Pierce™ IP Lysis Buffer (Thermo Fisher, USA). Lysates were kept on ice for 10 minutes and centrifuged at 12,000 rpm for 15 minutes. The supernatant was then incubated for 4 hours with protein A/G beads (Bimake, China) preloaded with EEF1D antibody (PTG, USA) at room temperature. The immunoprecipitate was rinsed three times with washing buffer (Thermo Fisher, USA), suspended in SDS loading buffer and boiled for 8 minutes at 95°C. Immunoprecipitated proteins were analyzed by SDS-PAGE/Western blotting.

### 2.9. Liquid Chromatograph-Mass Spectrometer (LC-MS) Analysis

In brief, immunoprecipitated peptides were applied for further liquid chromatograph-mass spectrometry (LC-MS) analysis using a micro HPLC (high pressure liquid chromatography) system connected to an LCQ Deca XP-plus ESI ion-trap mass spectrometer. Chromatographic separation is performed by normal-phase HPLC on a TSK-gel Amide-80 (3 *μ*m) column (2 × 150 mm). The LC-MS parameters were determined as described previously [15], and the mass spectra were obtained by ion monitoring based on *m*/*z*.

## 3. Results

### 3.1. Expression of EEF1D in LGG, GBM, and Nontumor Brain Tissues

**Figure 1:**
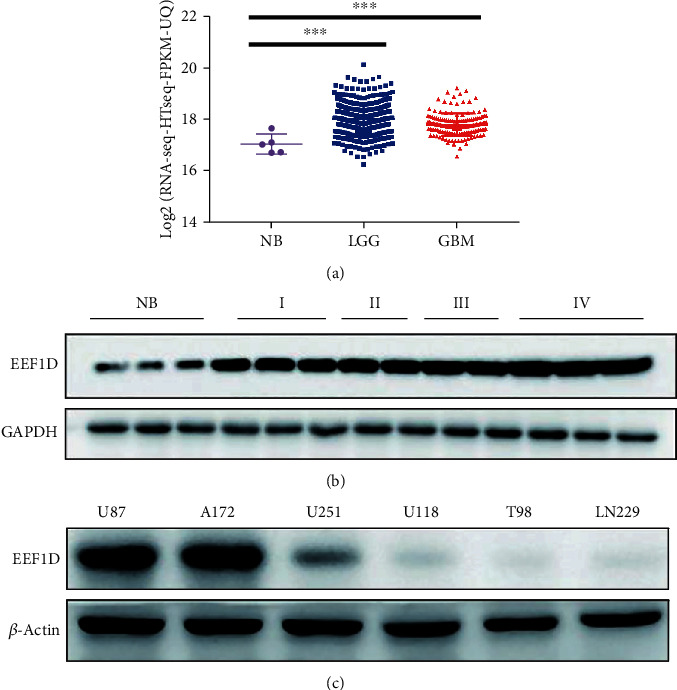
Expression of EEF1D in LGG, GBM, and nontumor brain tissues. (a) EEF1D expressions at mRNA level in normal brain tissues (*N* = 5), low-level glioma (LGG) (*N* = 511), and glioblastoma (GBM) (*N* = 156) were analyzed utilizing TCGA database. (b). EEF1D expressions at protein level were analyzed in clinical samples, including nontumor brain tissues (*N* = 3), grade I (*N* = 3), grade II (*N* = 2), grade III (*N* = 2), and grade IV (*N* = 3). (c). EEF1D expressions at protein level were analyzed in six glioma cell lines (U87, A172, U251, U118, T98, and LN229).

**Figure 2:**
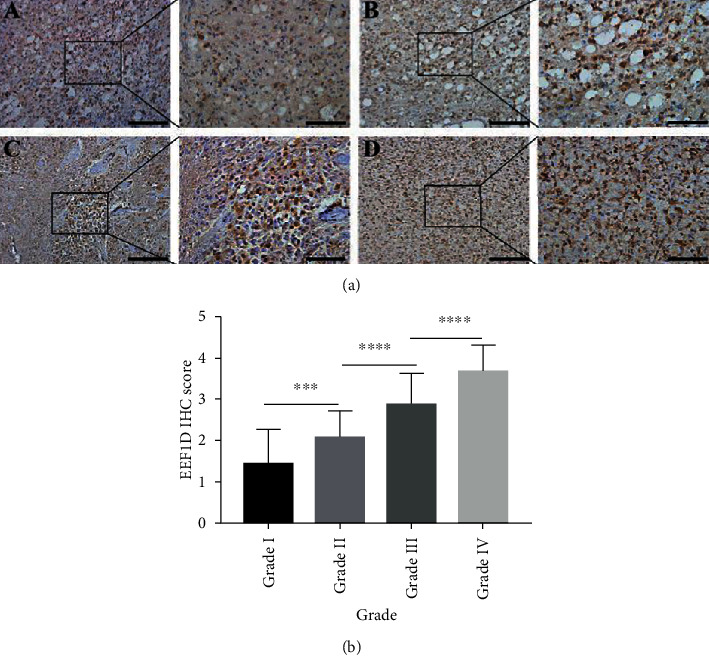
Expression of EEF1D in different grade glioma tissues by immunohistology. (a) Representative immunohistochemical staining samples of different grade human glioma (A—grade I, B—grade II, C—grade III, and D—grade IV) with anti-EEF1D antibody. Scale bars: 200 *μ*m for low magnification image and 100 *μ*m for high magnification image. (b) The IHC score of different clinical sample tissues (including grade I (*n* = 14), grade II (*n* = 38), grade III (*n* = 31), and grade IV (*n* = 25)) was analysed by using an independent samples *t* test (∗∗∗*P* < 0.001, *P* = 0.0043; ∗∗∗∗*P* < 0.0001).

By analyzing the data from TCGA database, we characterized EEF1D expression in 5 cases of nontumor brain tissues, 511 cases of LGG samples, and 156 cases of GBM samples. EEF1D expression was significantly higher in LGG and GBM samples (*P* < 0.01) (Figure 1(a)). Besides, consistent with TCGA results, EEF1D was increased in LGG and GBM compared to normal brain tissues by immunoblotting (Figure 1(b)). Consistently, we further confirmed higher expression in high-grade glioma than low-grade glioma using IHC staining and the IHC score (Figure 2). Moreover, we also detected higher EEF1D expression in U87 and A172 glioblastoma cell lines among six commonly used glioma cell lines (Figure 1(c)), so U87 and A172 glioblastoma cell lines were chosen for a further experiment.

### 3.2. Inhibition of EEF1D Impaired Proliferation, Migration, and Invasion of Glioma Cells

**Figure 3:**
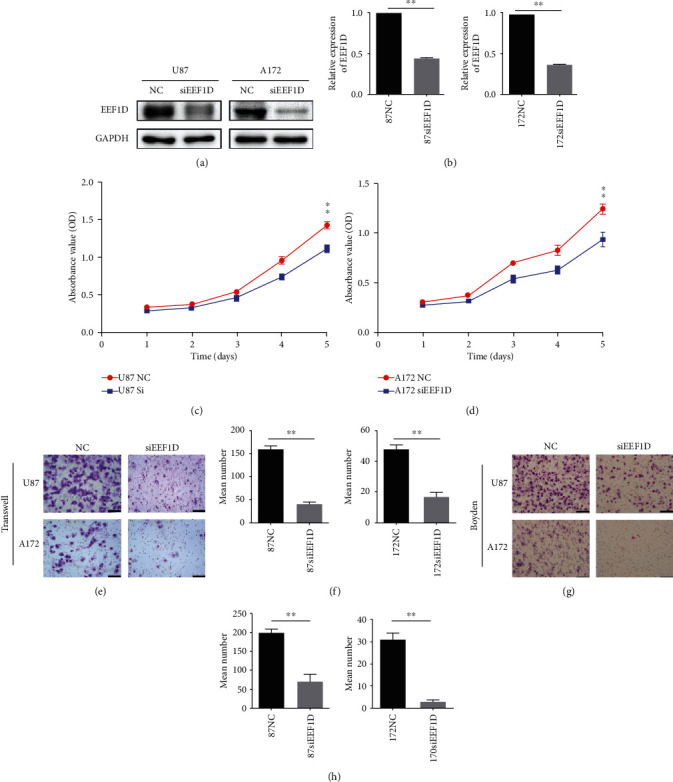
Inhibition of EEF1D impaired proliferation, migration, and invasion of glioma cells. (a) Efficacy of EEF1D-siRNA on EEF1D expression in U87 and A172 glioma cells, respectively. (b) Quantification results of (a). (c) Effect of EEF1D on proliferation of U87 and A172 glioma cells in vitro. The U87 and A172 glioma cells were subjected to CCK-8 assay for 5 d. There was a significant difference between two groups at day 5. NC: negative control siRNA-transfected cells; siEEF1D: EEF1D-siRNA transfected cells (^∗∗^*P* < 0.01 as compared with NC). (d) Quantification results of (c). (e, g) Transwell assay and Boyden assay were performed to determine cell migration and invasion in glioma U87 and A172 cell transfected with EEF1D-siRNA or negative control siRNA, respectively. NC: negative control siRNA-transfected cells; siEEF1D: EEF1D-siRNA-transfected cells (^∗∗^*P* < 0.01 as compared with NC). Scale bars: 100 *μ*m. (f, h) Quantification results of (c) and (e) respectively.

To investigate the influence of EEF1D on glioma cells, we constructed siRNA targeting EEF1D gene. As expected, EEF1D expression was significantly decreased in both U87 and A172 cells after transfection with EEF1D-siRNA (Figures 3(a) and 3(b)), which in turn resulted in significant inhibition of glioma cell proliferation (Figures 3(c) and 3(d)). Transwell assay further determined that depletion of EEF1D could impair the migration capacity of U87 and A172 cells (Figures 3(e) and 3(f)) and resulted in lower invasion capacity compared to negative control group, as Boyden assays have shown (Figures 3(g) and 3(h)). In summary, these results proposed that inhibition of EEF1D could suppress the cell growth and reduce the malignant phenotype-like proliferation, migratory, and invasion of glioma cells.

### 3.3. Knockdown of EEF1D Could Regulate the Expression of EMT Markers and Suppressed the Activation of PI3K/Akt Signaling Pathway

**Figure 4:**
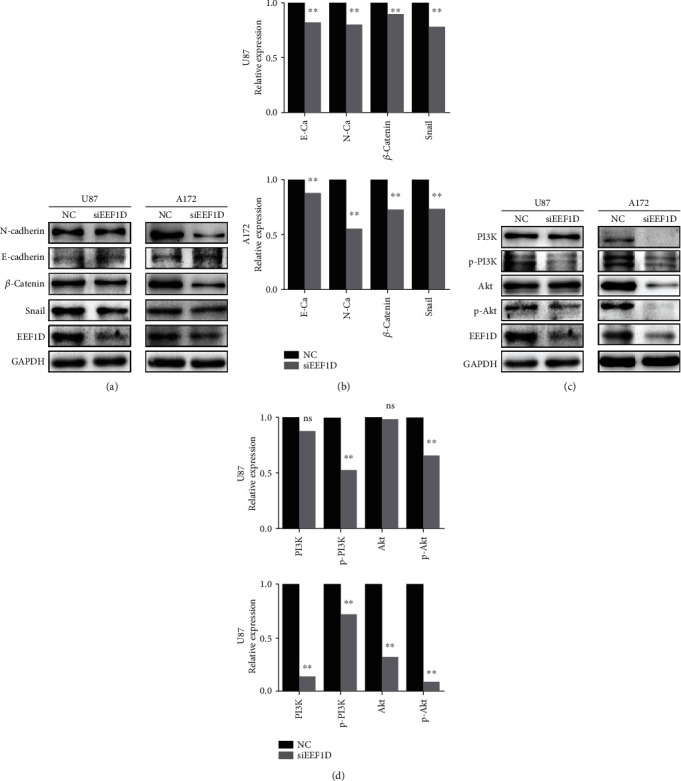
Downregulation of EEF1D could regulate the expression of EMT markers and suppressed the activation of the PI3K/AKT signaling pathway. (a) Protein levels of EMT markers were detected in U87 and A172 glioma cells by Western blot. GAPDH was also detected as the control of sample loading. NC: negative control siRNA-transfected cells. siEEF1D: EEF1D-siRNA-transfected cells. Data were based on at least three independent experiments and shown as the mean ± SD (^∗∗^*P* < 0.01 as compared with NC). (b) Quantification results of (a). (c) Protein levels of PI3K/Akt signal pathway were detected in U87 and A172 glioma cells by Western blot. GAPDH was also detected as the control of sample loading. NC: negative control siRNA-transfected cells; siEEF1D: EEF1D-siRNA-transfected cells. Data were based on at least three independent experiments and shown as the mean ± SD (^∗∗^*P* < 0.01 as compared with NC). (d) Quantification results of (c).

To investigate the underlying mechanism of EEF1D, the protein levels of EMT markers and PI3K/Akt signaling pathway were measured to further determine the effects of EEF1D suppression after transfection with EEF1D-siRNA in U87 and A172 glioma cells. As shown in Figures 4(a) and 4(b), levels of the epithelial marker E-cadherin were slightly promoted, whereas the expression of mesenchymal markers including N-cadherin and Snail was significantly downregulated in U87 and A172 glioma cells following EEF1D-siRNA transfection. Further, *β*-catenin, a critical transcriptional factor of the EMT process, was also decreased after EEF1D knockdown. Taken together, these findings suggested that EEF1D was closely associated with the modulation of EMT progress in glioma cells, and inhibition of EEF1D could reverse the EMT characteristics of glioma cells.

The PI3K/Akt pathway has been identified as a key regulator to growth and proliferation of malignant glioma cells. In the present study, Western blot results revealed that PI3K, p-PI3K, Akt, and p-Akt in glioma cells transfected with EEF1D-siRNA were significantly lower than those transfected with NC-siRNA (*P* < 0.05; Figure 4(c)). Thereby, the downregulation of EEF1D expression suppressed the activation of the PI3K/Akt pathway, which in turn might impede cell proliferation.

### 3.4. Identification of EEF1D Interacting Proteins

**Figure 5:**
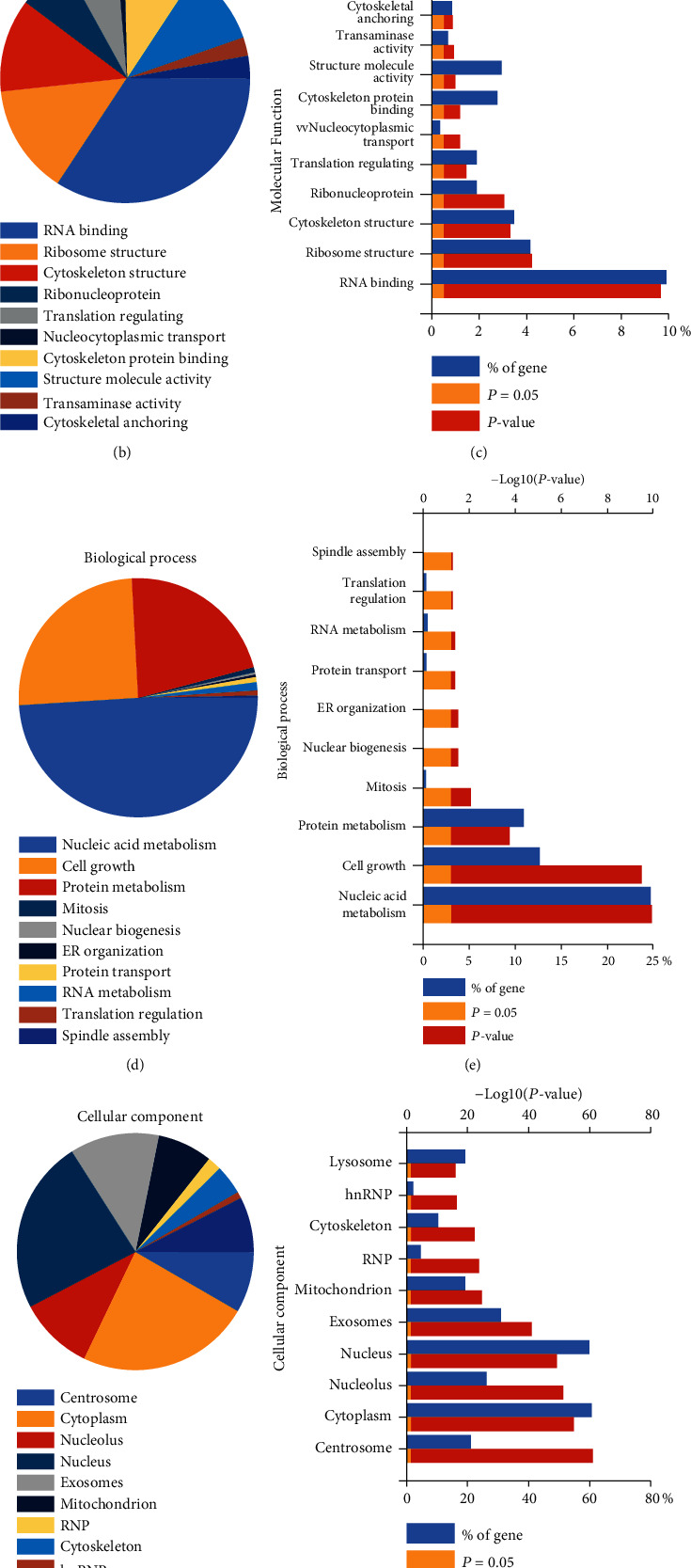
Identification of EEF1D interacting proteins. (a) Immunoprecipitation assays showing a good efficacy of specific EEF1D antibody. The top 10 enriched (b and c) molecular function (Venkatesan, Lamfers et al.) pathways, (d and e) biological process (BP) pathways, and (f and g) cellular component (CC) pathways of putative EEF1D-interacting protein from GO analysis by FunRich.

In order to further figure out the regulatory network of EEF1D on several signaling pathways, we carried out immunoprecipitation assays combined with LC-MS/MS to achieve qualitative and quantitative analyses of the potential EEF1D-interacting partners. As expected, EEF1D was immunoprecipitated with the EEF1D-specific antibody while no protein amounts were detected in the control IgG sample (Figure 5(a)). The top 20 putative specific interactors are listed in Table 1. Next, GO functional and KEGG pathway enrichment analyses were performed using the software of enrichment analysis tool FunRich: Functional Enrichment analysis tool [16–18]. Based on their primary biological processes, the majority of these proteins were considered involved in distinct functions. The detailed enriched GO functions for the interacting proteins are presented in Supplement Excel. In aspect of molecular function (Venkatesan, Lamfers et al.), top 10 associated functions were RNA binding, structural constituent of ribosome, structural constituent of cytoskeleton, ribonucleoprotein, translation regulator activity, et al., as displayed in Figures 5(b) and 5(c). Thus, the annotation of the biological process (BP) showed that proteins immunoprecipitated with EEF1D were mainly related to the regulation of nucleobase, nucleoside, nucleotide, and nucleic acid metabolism; signal transduction; cell growth and/or maintenance; and protein metabolism (Figures 5(d) and 5(e)). In addition, distribution of interacting proteins according to cellular component (CC) was also noted (Figures 5(f) and 5(g)). Via the biological pathway analysis, we discovered that the nucleic acid metabolism and cell growth were the most significantly enriched. Therefore, we speculated that EEF1D was likely to be involved in DNA replication and RNA synthesis through binding catalytic proteins and regulating its capacity.

Taken together, these results indicated that EEF1D might bind to other proteins, like the protein of the EMT and PI3K/Akt pathway, to form a functional complex to regulate the progression phenotype of glioma cells.

## 4. Discussion

In the present study, aberrant expression of EEF1D was confirmed in glioma samples in comparison with nontumor brain tissues. Besides, blocking the EEF1D restrained the critical EMT and PI3K/Akt pathway. Furthermore, GO functional and KEGG pathway enrichment analyses revealed that EEF1D might bind to other proteins to mediate nucleic acid metabolism and cell growth. These results indicate that EEF1D may play a critical role in glioma cell proliferation, migration, and invasion and acts as an oncogene in glioma.

Proteomic analysis shows that EEF1D is overexpressed in right-sided colon cancer [19] and correlates with the invasive status of Adriamycin-resistant variants of DLKP, a squamous lung and cancer cell line [20]. Genomic analysis in medulloblastoma also reveals that a higher EEF1D level was adversely associated with outcome [11]. Isadora et al. reported that overexpressed EEF1D leads to the modulation of proliferation via cyclin D1 and EMT and invasion in oral squamous cell carcinoma [6]. Consistently, in the present study, we found that the expression of EEF1D, as indicated both by immunohistology staining and immunoblotting, was elevated and positively correlated with glioma grade.

Besides the canonical role of EEF1D as a direct substrate for casein kinase 2 (CK2), an important regulator of cell cycle progression, apoptosis, and transcription [21, 22], EEF1D, could also participate in autoubiquitination and degradation by interacting with SIAH E3 ubiquitin protein ligase 1 (SIAH-1) [23]. Several researchers reported that gene amplification, genetic methylation, and posttranslational modification may play a role in the mechanisms of EEF1 superfamily proteins in different cancers [24].

Epithelial-mesenchymal transition (EMT), represented by remarkable morphological changes from an epithelial cobblestone phenotype to an elongated fibroblastic phenotype, provides a new insight into the potential mechanism for the glioma metastasis and invasiveness. EMT is characterized by a decreased expression of epithelial markers, such as E-cadherin, and an increased expression of mesenchymal markers, such as N-cadherin and vimentin, and transcription factors, such as slug, snail, and *β*-catenin [25–28]. EMT is a transcriptional process in which epithelial cells adopt mesenchymal properties by loss of cell-cell adhesion, acquisition of migratory and invasive properties, and loss of cell polarity, related to tumor initiation, invasion, metastasis to distant sites, and resistance to chemo- and/or radiotherapy [27, 28]. The PI3K/Akt signaling pathway, a well-known pathway in the regulation of tumorigenesis, tumorigenesis, is also activated in glioma [29, 30]. As an important intracellular signaling pathway, PI3K/Akt is known to be closely associated with the proliferation, migration, and progression of tumors, including glioma [31–33]. Besides, EMT pathways could cross talk with the PI3K/Akt/GSK3*β* pathway in glioma, through HIF-1*α*, PTEN, and the WNT/*β*-catenin pathways [28].

Our results suggested that EEF1D exerts its effect on glioma by promoting EMT and PI3K/Akt signaling pathways, which could be the potential mechanism by which EEF1D promotes tumor progression.

According to the results of GO analysis of the EEF1D interacting protein, the most enriched molecular functions were involved in the regulation of RNA binding, structural constituent of ribosome, nucleic acid metabolism, et al., which is crucial for maintaining the normal state of the cell metabolism process. RNA binding, structural constituent of ribosome, and nucleic acid metabolism were involved in the conventional DNA replication and RNA transcription process, which is extremely activated in tumor, like breast cancer, colon cancer, and glioma. Several potential targeted proteins of EEF1D, such as NFIC and RBMX, were reported to participate in the regulation of proliferation and EMT transition [25, 34, 35]. RBMX, also known as hnRNP G, was involved in various processes, like pre-mRNA splicing and posttranscriptional regulatory mechanism [36, 37], which were related to several vital processes of tumor initiation, progression, and metastasis. Alteration of the processes induced by aberrant expression of EEF1D may impair the indispensable nucleic acid metabolism and translational deregulation and finally impede tumor cell survival and progression including glioma. As for the biological process, multifarious cancer-related processes, such as nucleic acid metabolism, cell growth, protein metabolism, and mitosis, were the most common among the enriched pathways of the interacting proteins, consistent to the enriched results of the molecular function.

## 5. Conclusion

Overall, our results contribute to the knowledge of EEF1D effects on glioma and provide insights into the mechanism of glioma through mediating EMT and PI3K/Akt pathways. Therefore, blocking EEF1D could restrain EMT and PI3K/Akt activity resulting in the suppression of cell growth and tumor progression. However, the detailed mechanism of EEF1D on glioma progression phenotype remains elusive and needs further investigation. Overall, our data provide evidence that EEF1D might serve as a potential therapeutic target for glioma.

## Figures and Tables

**Table 1:** The putative specific interactors of EEF1D.

Accession	Gene name	Description	Coverage	Peptides	PSMs	Unique peptides	AAs	MW (kDa)	calc. pI
P35579	MYH9	Myosin-9, OS = Homo sapiens, GN = MYH9, PE = 1, SV = 4	45.86734694	123	334	106	1960	226.392	5.6
Q86UP2	KTN1	Kinectin, OS = Homo sapiens, GN = KTN1, PE = 1, SV = 1	48.78408254	76	162	76	1357	156.179	5.64
Q15149	PLEC	Plectin, OS = Homo sapiens, GN = PLEC, PE = 1, SV = 3	11.33646456	52	58	52	4684	531.466	5.96
A0A024RCN6	VARS	VARS, OS = Homo sapiens, GN = VARS, PE = 3, SV = 1	29.2721519	41	80	41	1264	140.387	7.59
A7BI36	RRBP1	p180/ribosome receptor, OS = Homo sapiens, GN = RRBP1, PE = 2, SV = 2	29.28571429	39	55	39	1540	165.649	8.97
V9HWE1	HEL113	Epididymis luminal protein 113, OS = Homo sapiens, GN = HEL113, PE = 2, SV = 1	54.72103004	30	75	30	466	53.619	5.12
A0A0D9SF53	DDX3X	ATP-dependent RNA helicase DDX3X, OS = Homo sapiens, GN = DDX3X, PE = 1, SV = 1	32.74215553	28	44	27	733	81.426	8.07
H6VRG1	KRT1	Keratin 1, OS = Homo sapiens, GN = KRT1, PE = 3, SV = 1	40.31007752	29	64	24	645	66.086	8.12
P35908	KRT2	Keratin, type II cytoskeletal 2 epidermal, OS = Homo sapiens, GN = KRT2, PE = 1, SV = 2	41.15805947	27	46	21	639	65.393	8
A0A024RBH2	CKAP4	Cytoskeleton-associated protein 4, isoform CRA_c, OS = Homo sapiens, GN = CKAP4, PE = 4, SV = 1	42.19269103	21	28	21	602	65.983	5.92
Q12965	MYO1E	Unconventional myosin-Ie, OS = Homo sapiens, GN = MYO1E, PE = 1, SV = 2	17.96028881	20	27	20	1108	126.982	8.92
Q92900	UPF1	Regulator of nonsense transcript 1, OS = Homo sapiens, GN = UPF1, PE = 1, SV = 2	17.00620018	19	25	19	1129	124.267	6.61
Q9C0C2	TNKS1BP1	182 kDa tankyrase-1-binding protein, OS = Homo sapiens, GN = TNKS1BP1, PE = 1, SV = 4	13.47599769	19	19	19	1729	181.685	4.86
P13645	KRT10	Keratin, type I cytoskeletal 10, OS = Homo sapiens, GN = KRT10, PE = 1, SV = 6	30.1369863	20	40	18	584	58.792	5.21
Q9UPQ9	TNRC6B	Trinucleotide repeat-containing gene 6B protein, OS = Homo sapiens, GN = TNRC6B, PE = 1, SV = 4	13.09328969	18	25	18	1833	193.883	6.76
Q1KMD3	HNRNPUL2	Heterogeneous nuclear ribonucleoprotein U-like protein 2, OS = Homo sapiens, GN = HNRNPUL2, PE = 1, SV = 1	22.75769746	18	23	18	747	85.052	4.91
P35527	KRT9	Keratin, type I cytoskeletal 9, OS = Homo sapiens, GN = KRT9, PE = 1, SV = 3	28.57142857	17	42	17	623	62.027	5.24
Q96PK6	RBM14	RNA-binding protein 14, OS = Homo sapiens, GN = RBM14, PE = 1, SV = 2	24.36472347	17	27	17	669	69.449	9.67
Q5JSZ5	PRRC2B	Protein PRRC2B, OS = Homo sapiens, GN = PRRC2B, PE = 1, SV = 2	10.31852849	18	23	17	2229	242.817	8.34
A0A087WTP3	KHSRP	Far upstream element-binding protein 2, OS = Homo sapiens, GN = KHSRP, PE = 1, SV = 1	30.09845288	17	28	17	711	72.982	7.71

## Data Availability

The genomic expression data and clinicopathological data of 163 GBM tissues, 518 LGG tissues, and 207 normal brain tissues were downloaded from The Cancer Genome Atlas (TCGA, https://tcga-data.nci.nih.gov/TCGA). The putative EEF1D-interacting proteins were subjected to functional annotation analysis using FunRich analysis tool (Pathan et al. 2015).
